# Determinants of Natural Mating Success in the Cannibalistic Orb-Web Spider *Argiope bruennichi*

**DOI:** 10.1371/journal.pone.0031389

**Published:** 2012-02-03

**Authors:** Stefanie M. Zimmer, Klaas W. Welke, Jutta M. Schneider

**Affiliations:** Zoological Institute and Museum, Biozentrum Grindel, University of Hamburg, Hamburg, Germany; UC Santa Barbara, United States of America

## Abstract

Monogynous mating systems (low male mating rates) occur in various taxa and have evolved several times independently in spiders. Monogyny is associated with remarkable male mating strategies and predicted to evolve under a male-biased sex ratio. While male reproductive strategies are well documented and male mating rates are easy to quantify, especially in sexually cannibalistic species, female reproductive strategies, the optimal female mating rate, and the factors that affect the evolution of female mating rates are still unclear. In this study, we examined natural female mating rates and tested the assumption of a male-biased sex ratio and female polyandry in a natural population of *Argiope bruennichi* in which we controlled female mating status prior to observations. We predicted variation in female mating frequencies as a result of spatial and temporal heterogeneity in the distribution of mature females and males. Females had a low average mating rate of 1.3 and the majority copulated only once. Polyandry did not entirely result from a male-biased sex-ratio but closely matched the rate of male bigamy. Male activity and the probability of polyandry correlated with factors affecting pheromone presence such as virgin females' density. We conclude that a strong sex ratio bias and high female mating rates are not necessary components of monogynous mating systems as long as males protect their paternity effectively and certain frequencies of bigyny stabilise the mating system.

## Introduction

Polyandry is a common phenomenon and requires an explanation as females in many species accept diverse costs of mating to gain more and/or more diverse sperm than required to fertilise their eggs [1]. Numerous studies measured benefits and costs of multiple mating to females but our understanding of the evolution of polyandry is still incomplete [2], [3], [4], [5]. It is assumed that benefits and costs of mating vary in magnitude, depending on how often a female mates [2]. Optimal female mating rates are likely to balance various costs and benefits of mating but natural mating rates will also reflect trade-offs between the degrees of competition, conflict, and cooperation within and between the sexes [2]. Optimal female mating rates should not be seen as fixed optima but as varying with different contexts e.g. defined by the degree of sexual conflict [2]. Females of some insect species, for example, are capable of modulating their mating rate depending on a set of environmental factors that affect the costs and benefits of mating [2], [6], [7], [8], [9]. Selection on male mating strategies may produce the side effect of removing the female mating rate from its optimum. In response, females will be under selection to develop adaptations that reduce the costs of mating and consequently shift the female mating rate back towards its optimum [10].

Reliable mating rates of wild animals are generally difficult to obtain and are often inferred from indirect evidence such as proximity of potential mating partners or courtship behaviour [11], [12], [13], [14]. However, such measures do not necessarily imply the successful fertilisation of eggs. Genetic methods used in the absence of field observations have the opposite problem, namely that it is unclear how many matings did not result in fertilisation. In invertebrates, quantitative field observations of copulation behaviour are particularly difficult to obtain, requiring high logistic effort. Rodriguez-Munoz et al. [15] determined natural mating rates through a combination of permanent video monitoring and subsequent DNA profiling. The results revealed a sex ratio very close to even in a wild population of field crickets, *Gryllus campestris*, and although male reproductive success varied more, both sexes had higher lifetime reproductive success when they had more mates.

In cases where only one method is used to measure mating rates, observed values are upper or lower estimates of actual values. Moreover, data obtained in laboratory studies need not necessarily be representative of natural conditions [2], [15], [16]. These short-comings and methodological difficulties may result in distorted views of actual mating rates and misjudgements of the operation of sexual selection and sexual conflict [17]. A well known example are the birds, in which genetic monogamy was generally assumed to be the prevailing mating system, until genetic evidence revealed that more than 70% of bird species perform extra-pair copulations [18].

In contrast to the classical concept that males should generally be selected to mate with multiple females [19], [20], it has recently been emphasised that males may sometimes be selected to invest strongly in paternity enhancement with one or two females only [21]. These monogynous (males fertilise a single female) or bigynous (males fertilise two females) mating strategies occur in several taxa and have evolved several times independently in spiders [21]. To increase their paternity with a single female, monogynous males evolved drastic mating strategies e.g. mate plugging by damaging genitalia in some *Argiope* species [22], or facilitation of sexual cannibalism by somersaulting onto the fangs of females as described in some *Latrodectus* species [23], [24]. The theory about the evolution of monogyny rests on the assumption of strong male competition for the fertilisation of females and the occurrence of a male-biased sex ratio combined with paternity benefits of increased mating investment [25]. The sex ratio bias has to result in different average mating rates of males and females to operate as a selection pressure on male monogamy [26], hence the traditional concept of the operational sex ratio (ratio of sexually active males to females; OSR) is not sufficient to describe this precondition as it does not relate to mating per se but to the potential for mating. An alternative concept is the effective sex ratio (ESR), defined as a ratio of males to females which includes only individuals that mate at least once [25]. The degree of male bias in the ESR defines the average male mating rate. A bias of two, for example, means that the average male mating success is 0.5. Under these conditions, a monogynous mutant that can secure fertilisation of more than 50% of a female's offspring has a mating success above average. The ESR can be male biased as a result of a biased tertiary sex ratio, or because a proportion of females do not mate (e.g. because they mature too late). Theory relies on polyandry as a precondition for evolution and maintenance of monogyny and bigyny [25], [26]. A mixture between monogyny and bigyny was found in *Argiope bruennichi*, but contrary to theory, effective mate plugging and avoidance of mated females by males suggest that most females mate only once [22], [27].

In the present study, we investigated natural female mating rates and tested the assumptions of theory with respect to the degree of polyandry and a bias in the ESR. We used a population of the sexually cannibalistic wasp spider *Argiope bruennichi*, a species with pronounced protandry and a highly seasonal breeding biology. Copulations may occur shortly after the female is moulting and are very brief and easily missed. To control female mating status at the onset of the observation, we removed penultimate females to moult in the laboratory. We returned the females to their exact position in the morning after their moult and monitored them closely until they got mated. Subsequently we regularly scan-sampled females until the end of the mating season.

We tested the hypothesis that more males than females would mate due to temporal and spatial variation in female mating rates. We predicted high female mating rates early in the season when protandrous males have matured already and the OSR is male biased. Furthermore, we predicted higher mating rates in female clusters that may attract more males. Correspondingly, we expected that late maturing females would remain unmated as well as females located at the edges of the population. In addition, we measured female body mass which is a proxy of fecundity and may cause variation in female mating rates as well.

## Materials and Methods

**Figure 1:**
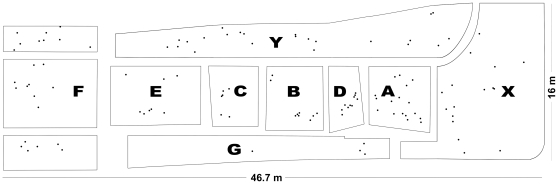
Map of the study field with nine distinct patches (A–G, X, Y) of different sizes which were separated from each other by narrow paths. The whole field contains a size of 747.2 m^2^. Dots indicate the location of individual female spiders in their webs which were marked and observed during the mating season. Females rarely changed their web sites.

The field study took place from July 2009 until August 2009. The study site was open grassland in Hamburg (permission was given verbally by the owner) bordered by unsuitable habitat on all sides (highway, road and gravel). The meadow had a shallow slope to one side and the density of the vegetation decreased down the slope. The whole field (747.2 m^2^) was divided into nine patches of different sizes, which were separated from each other by narrow paths (Fig. 1). These paths and uncovered areas within each patch were used to step on by the examiners to minimise destruction of webs and web supporting vegetation.

The study started after the first female had matured. Each immature female web location was marked with a bamboo pole and received an individual number. Additionally, the distance to the nearest female neighbour was measured and the female neighbours within a radius of 1 m, 2 m, 3 m and 5 m were counted. Every day, we moved carefully through the field and noted the number of males staying close to subadult females' webs or in their web.

During daily controls, the developmental status of each penultimate female was closely monitored. The swelling of the external genital structure of female spiders (epigyne) can be used to forecast the date of their final moult. When females are freshly moulted to sub-adulthood no swelling is present whereas close to final moult the swelling looks like that of an adult female but is still covered with a cuticular layer. Based on the epigyne swelling females were categorised in seven groups, the last of which were about to mature. In order to ensure that all copulations could be observed, penultimate females were removed from the study site as soon as they reached the last category. The removal occurred with great care not to damage the web or interfere with the surroundings. Thereby, we intended to minimise disturbance for males that were already present near the web. The categorisation ensured that we collected subadult females as shortly as possible before their final moult. In the laboratory, the collected subadult females were kept in individual Perspex frames (36 * 36 * 6.5 cm), where they built characteristic orb-webs. All spiders were fed with ca. 20 *Drosophila* spec. and one *Calliphora* spec. and sprayed with water once per day. In the morning after females moulted to maturity, which occurred within 3.56±1.55 days after capture, they were weighed on an electronic balance to the nearest 0.001 mg and they were individually marked with a unique combination of colour dots on their opisthosoma. Then the females were returned with the frame to the original web site and the frames were attached to the ground with tent pegs. The first adult female was released on the 10^th^ of July, the last one on the 7^th^ of August 2009. The interval between female removal and maturation did not affect female mating rate (monogamous or polygamous) (Logistic regression: χ^2^ = 0.51, p = 0.47, n = 98) nor the time required until copulation occurred (*delay factor*; r = −0.07, p = 0.48).

Previous studies on this and other spider species found that most matings happened just after the female's final moult [27], [28]. The females were observed for eight hours per day, which corresponds to the largest part of the available daylight period. If a virgin female did not copulate on a given day, she was carried back in her frame to the laboratory and released again the following day(s), until she mated. After having mated at least once, females were left in the field so as to maintain a natural occurrence of virgin and mated females. Based on previous field observations [27], we assumed that the attractiveness of females rapidly declines after copulation, because matings with mated females are mechanically difficult due to the presence of mating plugs and the expected paternity gain is close to zero [22]. When a male visited a female, we noted the following: his size and number of legs; the time of his first appearance; the times of his first contact with the frame, the web, and the female; the beginning and duration of courtship and copulation; the number of copulatory insertions; and the occurrence of sexual cannibalism or male escape from a female attack.

When males survived the copulation and left the web, they were captured and marked with a specific colour dot (with non-toxic PELIKAN Plaka paint) on their opisthosoma before they were released again. In this way they could be identified if they reappeared. After sexual cannibalism, the wrapped males were carefully taken out of the females' web and were frozen at −80°C in the freezer in the laboratory for further measurements.

Overall, 121 females were recorded and observed at the study site over the whole mating season. Of these, we excluded 11 females whose mating status was unclear because they were already adult when found. One subadult and one adult female disappeared in the laboratory and eight adult females escaped in the field before their mating rate could be measured. The mating of one virgin female in the field was missed and the last virgin female during the mating season did not get mated. Hence, mating rates of 98 females could be determined.

Data analyses were carried out with JMP 7.0.2. Most data were analysed using logistic regressions. Not normally distributed data were analysed with the non-parametric Wilcoxon Signed-Rank test and these tests are indicated with the results. We used an ANOVA to test the dependent spatial and temporal variables of male visitors' accumulation on females' web, as well as a multiple regression for the dependent variables on male activity. Descriptive statistics are given as mean ± standard error of the mean (SE) unless stated otherwise and sample sizes may differ between analyses due to missing data.

## Results

### Mating rates

On average, the 98 observed females attracted 2.61±0.23 visitors (range 0–14) that at least reached the frame in which the female had her web; only one female had no visitor and remained unmated. However, 66 males (on average 0.8 males per female) left the female web without proceeding to courtship and copulation. Because *A. bruennichi* males may use either one or both of their pedipalps with the same female, we distinguish a female's mating rate (defined as the number of males that performed at least one copulatory insertion with this female) from her copulation rate (defined as the sum of copulatory insertions performed with this female by any of her mates).

The average mating rate of females was 1.30±0.05 (0–4 range) and based on 98 females, of which 72 females (73.5%) mated with only a single male, 24 females (24.5%) mated with two males, one female (1%) mated with three males, and one female (1%) mated with four males.

We could not determine the final copulation rate for five females. We just found dead males in the webs of these five females and we did not know if they had copulated once or twice with the females before they were killed. Hence, copulation rate is known for 93 of the 98 females. The average copulation rate of females was 1.38±0.07 (0–4 range); 63 females (67.74%) copulated once, 24 females (25.81%) copulated twice, four females (4.3%) copulated three times, and only two females (2.15%) copulated four times. Of the 24 females that copulated twice, 15 females copulated once with each of two different males, whereas nine females copulated twice with the same male. No differences were detected (Binomial: χ^2^ = 1.52, p = 0.22, n = 24). Four females achieved their copulation rate of three through a single copulation with one male and a double copulation with another male. Only two females had four copulations; one through single copulations with four different males and the other one through double copulations with two different males.

The effective sex ratio (ESR), defined as the ratio of males to females that mate at least once [25], was 1.29 resulting from 98 females and 126 males that met the definition. The male bias may in fact be lower and the true ESR closer to even for the following reasons: The number of females may be higher because bigynous males were counted once although the 1^st^ mates of 22 (17.5%) bigynous males were unknown. Furthermore, we did not account for the number of females that only received a copulation from a bigynous male which could have inflated the number of males that mated and the male bias in the ESR would indeed be reduced. According to theory [25], this relatively weakly biased ESR implies that monogyny will be favoured by selection if and only if monogynists' expected paternity share is >77.5%.

For further analyses we group females in two categories concerning their mating success (the number of mates a female had during the entire mating season): those that received one mating and those that received more than one mating. Female mating success was not a function of female adult weight (Logistic regression: χ^2^ = 0.31, p = 0.58, n = 98). Below we analyse whether female mating success was influenced by spatial and temporal factors.

### Spatial patterns

Females were scattered over the whole study site although there were several small clusters (Fig. 1) so that nearest neighbour distances varied (see Material S1). The local density, measured as the number of other females in a radius of 1–5 m or as the distance to the nearest neighbour regardless of their mating status, did not correlate with the number of male visitors nor with the copulations that a female received (Table 1). However, females were less likely to mate with more than one male if the number of virgin females in the population was relatively high (Logistic regression: χ^2^ = 4.48, p = 0.03, n = 98).

**Table 1:** Correlations between female copulation frequencies or the number of male visitors per female with different measures of female density.

variable	with variable	*r*	n	p
Copulation frequency per female	**♀♀** in 1 m radius	−0.11	98	0.3
	**♀♀** in 2 m radius	−0.1	98	0.34
	**♀♀** in 3 m radius	−0.11	98	0.3
	**♀♀** in 5 m radius	−0.14	98	0.16
	distance of nearest neighbours	−0.03	98	0.77
Male visitors per female	**♀♀** in 1 m radius	0.12	109	0.23
	**♀♀** in 2 m radius	−0.04	109	0.65
	**♀♀** in 3 m radius	0.002	109	0.99
	**♀♀** in 5 m radius	−0.01	109	0.89
	distance of nearest neighbours	−0.07	109	0.48

Notably, many males waited in close proximity to subadult females, either in their own webs or in the outer region of a female's web. Usually in such cases there were one or two males (in one case even three males) associated with a given subadult female. The number of males waiting at a female's web depended on the female's developmental state. Females close to their final moult attracted more males (0.77±0.14; n = 26) than females that were more than a week away from adulthood (0.2±0.11 males; n = 21; Wilcoxon Signed-Rank test: χ^2^ = 9.7, p = 0.002). In order to avoid pseudoreplication through repeated sightings of the same males we analyzed this pattern further using a single day of the mating season. We picked day six because on that day the ratio of subadult females to adult males was balanced. On this day, the number of males at a subadult female's web was significantly predicted by an ANOVA (F_14,35_ = 4.62, r^2^ = 0.65, p = 0.0001) with Patch ID (F_8,41_ = 3.61, p = 0.004) and female developmental state (F_6,43_ = 7.48, p = <0.0001) as factors.

### Temporal patterns

**Figure 2:**
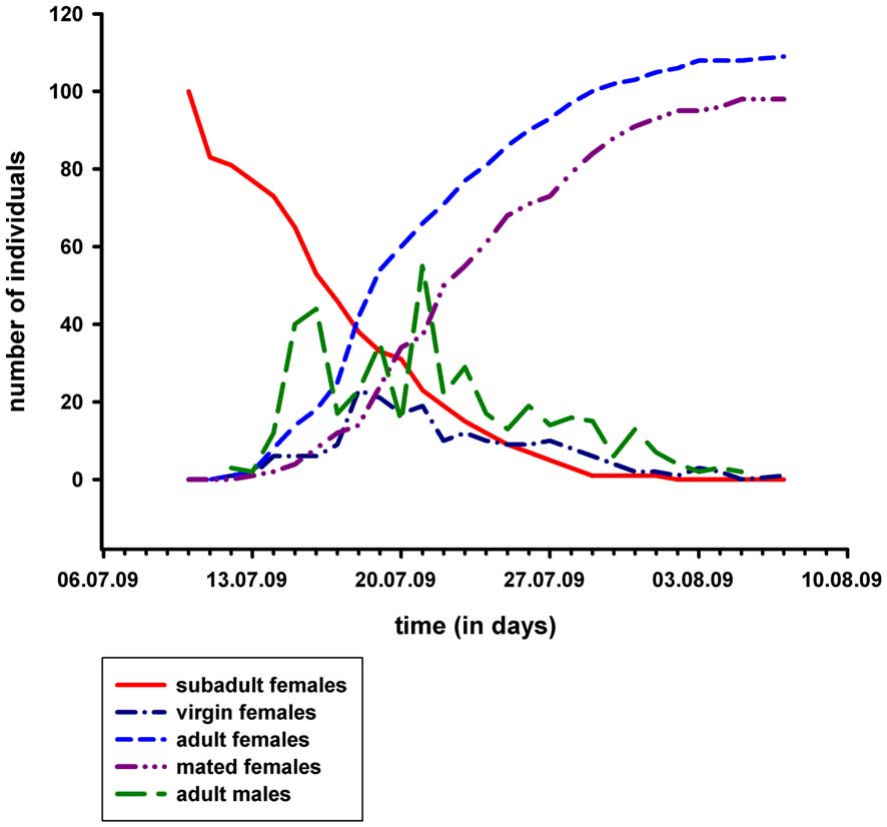
Daily numbers of subadult (red line) and adult females (blue dashed line) and adult males (green dashed line) in the population. Adult females are also separated according to their mating status (virgin females (dark blue dashed line) and mated females (purple dashed line)). The mating season started at the 12^th^ of July with the 1^st^ adult virgin female.

**Figure 3:**
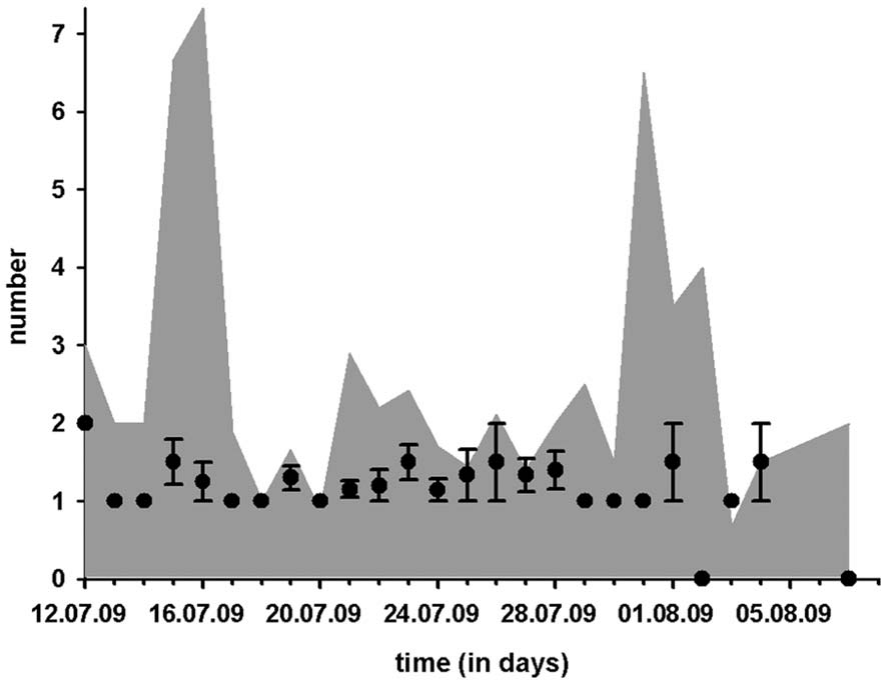
The grey area shows the ratio of adult males per virgin female calculated for each day of the season. The ratio is generally male biased with two distinct peaks early and late in the season. The dots depict daily female mating rates. If more than one female mated on a particular day, mean and SE of mating rates are given.

The mating season lasted 30 days (see Fig. 2). Accordingly, the daily availability of virgin females was highest during the first eight days, reached a peak on the 18^th^ of July with 23 freshly moulted females and then declined: 50% of the females moulted to maturity during the first week. Roving males were seen until the 6^th^ of August and the daily counts fluctuated during the first two weeks of the season and steadily declined during the last two weeks. The number of roving males was always higher than the number of virgin females and showed two major peaks, one early and one later in the season (Fig. 3). These peaks in male activity were not reflected in daily female mating rates which varied between one and two throughout the course of the season (Fig. 3). Accordingly, the variation in female mating rates (female mating rates are categorized as “one” or “larger than one”) was not explained by the number of active males (Logistic regression: χ^2^ = 0.97, p = 0.32, n = 98) nor by the day in the season on which the females matured (Logistic regression: χ^2^ = 0.04, p = 0.85, n = 98).

Fluctuations in male activity, measured as the number of male visitors per female per day, were significantly explained by how many virgin females were present in the population (F_1,24_ = 4.58, p = 0.0001) but not by the day of the season (F_1,24_ = 0.65, p = 0.52; multiple regression: F_2,22_ = 11.1, r^2^ = 0.5, p = 0.0005). Hence, high numbers of virgin females increase male activity but decrease female mating rates (see above).

### Female attractiveness

All females except one received at least one copulation but not every female was mated soon after she appeared on the meadow. In fact, only 17.4% of the females received a copulation within the first two hours of their release. Many females (n = 55) had to be repeatedly released on up to five consecutive days. We categorised females' mating latency as ≤2 h or >2 h. Females in these two categories did not differ significantly in any of the following variables: day of first (Logistic regression: χ^2^ = 0.33, p = 0.57, n = 98) and last release (Logistic regression: χ^2^ = 2.0, p = 0.16, n = 98); female size (Logistic regression: χ^2^ = 2.87, p = 0.09, n = 98); and nearby female density in the field (Logistic regression: 1 m radius: χ^2^ = 0.003, p = 0.95, n = 98; 2 m radius: χ^2^ = 0.002, p = 0.97, n = 98; 3 m radius: χ^2^ = 0.007, p = 0.93, n = 98; 5 m radius: χ^2^ = 0.05, p = 0.83, n = 98; nearest neighbour distance: χ^2^ = 0.65, p = 0.42, n = 98). 40.7% of these females (33 of 81 females) were actively rejected by visiting males (20 by one male, 13 by several males), while 59.3% simply had no visitors prior to their first copulation. Rejected females did not differ from unvisited females in the above parameters (all p>0.05).

## Discussion

Contrary to our predictions about the mating system of *Argiope bruennichi*, we found surprisingly low variance in female mating rates over the course of the season. Polyandry was rare and female mating rates were not related to temporal and spatial homogeneity in the distribution of mature females and males. The small variance in female mating rates is likely explained by life-history and mating decisions of males and it remains unclear whether observed mating rates are merely male imposed or in which degree they reflect female interests.

Many orb-weaving spiders, including all species of the genus *Argiope*, are characterised by a pronounced sexual size dimorphism and small male size is generally explained by selection on protandry [29], [30]. Protandry means that males mature before females and if females mature progressively, a dramatic shift in the operational sex ratio is expected from an early male bias to a late female bias. The latter is expected if males are removed from the mating market for example through sexual cannibalism [31], [32] which is the case in our study system. As a consequence of this pattern, we expected strong competition among protandrous males over the earliest maturing females and an elevated mating rate in these females. High rates of polyandry of early females and low mating rates of males may have the effect that late maturing females remain unmated because no males are available any more. However, we did not find the expected pattern and only a single female did not mate. Males were found active throughout the season and there was even a second peak of male abundance during the second half of the season, strongly opposing the idea of protandry. This finding suggests that males keep maturing at least during the first half of the season and/or that males survive long enough to wait for opportunities to copulate with virgins. Below we discuss male life-history decisions and male choice as possible explanations for the observed pattern of low female mating rates and low variance in female mating rates.

Males of the sexually cannibalistic spider *L. hasselti* were shown to change their development time in response to pheromones that provide cues of female and male density. Without female odour cues, males delayed maturation in favour of reaching a better condition to survive mate searching and competition for sparse females. With female odour cues, males speeded up development and matured smaller and in poorer condition [33]. As suggested for several further species with similar mating systems [33], males of *A. bruennichi* may also be able to flexibly truncate or prolong the penultimate instar and adjust the timing of maturation to the presence of pheromone cues emitted by females in the vicinity. As the local presence of volatile cues varies over time, such a mechanism could lead to the continuous maturation of males as long as virgin females mature. Although pheromone dependent maturation has not yet been studied in *A. bruennichi*, the potential is present as males of this species are known to adjust their mating strategy to the past perception of female sex pheromones [34].

The perception of female availability and female mating status through volatile pheromones provided the base for our prediction of spatial variation in mating rates. As female webs in high density patches should attract more males we expected higher mating rates. However, although more female cues were likely present in high densities, this did not result in elevated rates of mating or male visitation in this study. In contrast, a study on *Nephila plumipes* showed that females formed clusters between which the opportunity of males to find suitable mates differed [35]. In some areas, males encountered many females and accumulated on female webs, probably leading to increased female mating rates. In contrast, other locations were difficult to find for males and consequently females in these patches were never reached by males and remained unmated. In comparison to *Nephila*, the distribution of *Argiope* species is generally less patchy. Females may occur in small clusters but distances between clusters are short [36].

Low incidences of polyandry and the absence of a correlation between the number of male visitors and female mating rates may be a result of male discrimination against mated females [27]. Avoiding mated females appears highly adaptive in view of the paternity protection mechanisms in *A. bruennichi*: mated females usually have at least one of their two genital openings plugged with a genital sclerite of the first male to mate [22]. Such mating plugs are highly effective, and although most males inseminate and plug only one side, a following male apparently cannot detect which side is virgin [22]. Hence, a male that copulates with a half-mated female has a 50% risk of using a plugged side. If sperm transfer fails in this case and the male falls victim to sexual cannibalism, he will not have any reproductive success. The finding that mated females appear to be relatively unattractive raises the question of whether extreme male mating strategies such as self-sacrifice and genital damage are really necessary to protect a male's paternity.

In accordance with an earlier field study [27], some males even abandoned virgin females after a brief inspection of the web. The majority of freshly moulted virgin females had to be taken to the field several times before they received a copulation. While one half of the females were not visited by males, 40.7% were actively rejected by a visiting male at least once before they received a copulation. However, female mating latency was not related to her size, the time in the season nor to female density. Female mating success was lower if more other virgin females were present, suggesting that males were more likely to leave a virgin if alternative mating opportunities were available and that females compete for mates. Females are known to emit male-attracting pheromones [37] and variation in the quantity of such pheromones may explain why male activity in our study correlated with the number of virgin females. Experimental tests with varying pheromone dosages are required to substantiate this speculation.

Pheromones are mostly found on the webs of virgin females [38] suggesting that males perceive a female only after or during the moult to maturity. However, our observations are in accordance with reports from *A. aurantia* [39] in which males position themselves in the vicinity of females that are about to mature. Thereby they likely increase the probability to mate first with a virgin but the competition with the other males surrounding the same female may be severe.

By removing subadult females (along with any associated sensory cues) from the males for a few days, we may have interfered with male mating strategies. The time between the removal of the subadult female and the returning of the freshly moulted adult varied but the length of this time period did not influence her mating rate or mating latency. Even though we made sure to leave the web and the surrounding vegetation intact, we cannot exclude that female removal had an effect. At worst, this manipulation may have increased male mortality and influenced the sex ratio so that we underestimated female mating rates. Even though we would not expect major distortions, future studies should account for male guarding of penultimate females and simulate this situation to assess whether it provides a fundamentally different scenario. In addition, the nature of the cues used by males to identify subadult females close to maturity needs to be investigated. Perhaps pheromones are already present at this stage, at dosages too low to be detected by gas chromatography-mass spectrometry [38], but high enough to be detectable by male spiders. Alternatively, there might be an additional volatile substance that is used as a cue for female's reproductive status, receptivity, and attractiveness.

Theory suggests that a monogynous male mating strategy can evolve when two conditions are met. First, monogynous males should possess a mechanism of paternity protection not available to polygynous males [25], [26]. This is true for *A. bruennichi*, in which monogynous males have the potential of monopolising a female by leaving mating plugs in both of her genital openings [22], [40]. Second, there should be a male-biased effective sex ratio (ESR; based on males and females that mate at least once). The male bias in the ESR is required if the high investment in monopolisation is to elevate male mating success above the average.

However, our data reveal only a weak bias of 1.29 in the study population which then requires a very potent paternity protection with a success of over 77%. Otherwise a monogynous mating strategy would not be better than polygyny. Indeed, plugging success and effectiveness are very high (above 80% in the laboratory) in *A. bruennichi* [22].

Female mating rates were unexpectedly low but still above one. The proportion of doubly mated females was partly explained by the proportion of bigynous males [Welke, Zimmer, Schneider, unpublished]. Bigynous males escape sexual cannibalism after their first mating and search for a new female instead of re-mating with the same one. The presence of bigynous males elevates female mating rates and increases the need for paternity protection even under an even sex ratio and male discrimination against mated females. At the same time, bigynous males will lower the ESR.

A potential source of error may have caused an underestimation of female mating rates. Mated females remained in the field and may have received additional copulations that took place undetected, e.g. because they occurred at night. However, field observations of focal females at night *A. bruennichi* suggest that copulations rarely if at all occur at night (SMZ, personal observation). Nevertheless, 22 unmarked mated males were found which means that their first copulation was missed. This strongly implies that a proportion of copulations occurred either outside the observation times or outside the observed population. These unseen 1^st^ copulations could have occurred with virgin or mated females. The latter case would not affect the ESR while unnoticed once-mated females would reduce the male bias in the ESR. Hence, our main conclusions may be robust with respect to unnoticed copulations.

Overall, our results suggest a weak ESR bias in *A. bruennichi* and support the theoretical prediction that even a weak bias is sufficient to favour monogyny given that monogynists possess efficient mechanisms of paternity protection [25]. On the other hand, as the frequency of monogyny increases and monogynists compete increasingly with each other, the fitness of this strategy decreases, potentially leading to a mixture of mono- and bigynous mating strategies that is maintained by negative frequency-dependent selection [26]. Since any increase in the frequency of bigynous males would necessarily elevate the degree of polyandry and hence the need for paternity protection, it seems likely that the coexistence of monogyny and bigyny stabilised the mating system.

To our knowledge, this study provides the first study design to examine natural female mating rates in a population of orb-web spiders. Further studies on different populations, and on other species, are required to fully understand the selection factors that have shaped the remarkable mating system of this and other species.

## Supporting Information

Material S1 **Nearest neighbour distances in metre between females.**
(DOC)
