# Supplementary material for: Determinants of Natural Mating Success in the Cannibalistic Orb-Web Spider Argiope bruennichi
Source: PLoS One. 2012 Feb 3;7(2):e31389. doi: 10.1371/journal.pone.0031389 (PMC3272030; doi:10.1371/journal.pone.0031389)
Supplement: Material S1 — Nearest neighbour distances in metre between females. (DOC) [file pone.0031389.s001.doc]

| **Female ID** | **Patch** | **Nearest Neighbour Distance in metre** |
| --- | --- | --- |
| **A2** | **A** | **1,198** |
| **D1** | **D** | **0,22** |
| **C5** | **C** | **0,156** |
| **Y14** | **Y** | **1,26** |
| **X4** | **X** | **2,19** |
| **F4** | **F** | **1,31** |
| **A1** | **A** | **0,87** |
| **C1** | **C** | **0,625** |
| **D2** | **D** | **0,37** |
| **X5** | **X** | **3,675** |
| **F2** | **F** | **0,625** |
| **F5** | **F** | **0,49** |
| **D6** | **D** | **0,22** |
| **X2** | **X** | **1,25** |
| **Y4** | **Y** | **0,81** |
| **Y6** | **Y** | **0,44** |
| **X1** | **X** | **0,19** |
| **A3** | **A** | **0,37** |
| **B3** | **B** | **0,2** |
| **E2** | **E** | **0,5** |
| **B1** | **B** | **0,19** |
| **D4** | **D** | **0,218** |
| **A4** | **A** | **0,531** |
| **B4** | **B** | **0,55** |
| **Y1** | **Y** | **1,25** |
| **X6** | **X** | **1,25** |
| **F1** | **F** | **0,25** |
| **F3** | **F** | **0,625** |
| **A5** | **A** | **0,375** |
| **C2** | **C** | **0,625** |
| **A6** | **A** | **0,37** |
| **F7** | **F** | **1,1** |
| **Y8** | **Y** | **0,87** |
| **A7** | **A** | **0,813** |
| **X7** | **X** | **2,2** |
| **D3** | **D** | **0,375** |
| **Y2** | **Y** | **1,1** |
| **Y3** | **Y** | **0,68** |
| **E1** | **E** | **0,5** |
| **X3** | **X** | **0,755** |
| **F6** | **F** | **0,25** |
| **F9** | **F** | **0,9** |
| **Y7** | **Y** | **0,68** |
| **E4** | **E** | **1,34** |
| **D7** | **D** | **0,28** |
| **B2** | **B** | **0,49** |
| **E6** | **E** | **3,75** |
| **F12** | **F** | **0,84** |
| **Y11** | **Y** | **0,25** |
| **B5** | **B** | **0,55** |
| **E5** | **E** | **0,25** |
| **F11** | **F** | **3,19** |
| **X9** | **X** | **0,875** |
| **X8** | **X** | **0,55** |
| **X10** | **X** | **0,19** |
| **C3** | **C** | **0,72** |
| **Y10** | **Y** | **0,375** |
| **Y9** | **Y** | **1,28** |
| **X11** | **X** | **0,55** |
| **A9** | **A** | **0,531** |
| **C4** | **C** | **0,156** |
| **F8** | **F** | **0,9** |
| **Y12** | **Y** | **0,187** |
| **Y13** | **Y** | **3,76** |
| **A8** | **A** | **0,813** |
| **F16** | **F** | **1,22** |
| **F17** | **F** | **0,78** |
| **D5** | **D** | **0,219** |
| **E3** | **E** | **1,38** |
| **F15** | **F** | **0,66** |
| **F13** | **F** | **0,84** |
| **B6** | **B** | **0,19** |
| **Y15** | **Y** | **0,78** |
| **A10** | **A** | **0,75** |
| **X13** | **X** | **1,4** |
| **X12** | **X** | **2,85** |
| **D8** | **D** | **0,5** |
| **A11** | **A** | **0,49** |
| **E7** | **E** | **1,38** |
| **X15** | **X** | **2,2** |
| **F14** | **F** | **1,31** |
| **B7** | **B** | **0,25** |
| **F20** | **F** | **0,69** |
| **A13** | **A** | **1,16** |
| **X14** | **X** | **0,755** |
| **F19** | **F** | **0,69** |
| **G1** | **G** | **0,63** |
| **Y17** | **Y** | **0,78** |
| **Y16** | **Y** | **0,875** |
| **F18** | **F** | **0,66** |
| **E8** | **E** | **0,25** |
| **Y18** | **Y** | **0,44** |
| **F10** | **F** | **0,49** |
| **F22** | **F** | **0,78** |
| **A12** | **A** | **0,313** |
| **Y19** | **Y** | **3,76** |
| **F23** | **F** | **1,22** |
| **C7** | **C** | **0,72** |
| **A14** | **A** | **0,375** |
| **X16** | **X** | **1,4** |
| **Y21** | **Y** | **0,375** |
| **Y20** | **Y** | **0,187** |
| **G2** | **G** | **0,84** |
| **Y22** | **Y** | **0,87** |
| **F21** | **F** | **3,19** |
| **G3** | **G** | **0,63** |
| **A15** | **A** | **0,81** |
| **F24** | **F** | **2,125** |
| **Y5** | **Y** | **0,875** |
| **D9** | **D** | **0,218** |
| **Y23** | **Y** | **1,25** |
| **B8** | **B** | **0,49** |
| **B9** | **B** | **0,55** |
